# Melodic bridges: music intervention as a catalyst for social skills development in preschool children with autism

**DOI:** 10.3389/fpsyg.2025.1542662

**Published:** 2025-06-25

**Authors:** Jinjin Yang, Runyi Zhang

**Affiliations:** ^1^College of Music, Yeungnam University, Daegu, Republic of Korea; ^2^College of Physical Education, Yeungnam University, Daegu, Republic of Korea

**Keywords:** music intervention, social skills, autism spectrum disorder (ASD), preschool children, therapeutic music activities

## Abstract

Autism spectrum disorder (ASD) is characterized by persistent challenges in social communication, emotional regulation, and cooperative behavior. While traditional behavioral therapies are widely used, they can lack engagement and flexibility. Music interventions, by engaging multiple neural and emotional systems, offer a promising alternative to address these core deficits. This randomized controlled trial (RCT) evaluated the efficacy of a 12-week structured music intervention in improving social skills in preschool children with ASD. Sixty participants (aged 3–6 years) were randomly assigned to an experimental group (music intervention) or a control group (behavioral therapy). Therapeutic music activities included rhythm training, interactive singing, instrumental improvisation, and group games, conducted three times weekly. Social skills were assessed using the Social Responsiveness Scale (SRS) and observational data at baseline (T0), mid-intervention (T1), post-intervention (T2), and six-week follow-up (T3). The music intervention demonstrated significant improvements in social communication, emotional regulation, and social motivation compared to the control group. Interactive singing showed the strongest impact on social communication, fostering verbal reciprocity and turn-taking. Rhythm training enhanced social motivation and joint attention, while instrumental improvisation improved emotional regulation by providing a non-verbal outlet for self-expression. Group games facilitated peer interaction and cooperation. These improvements were sustained at T3, underscoring the intervention’s durability. This study highlights the transformative potential of music interventions in addressing core social deficits in preschool children with ASD. By leveraging rhythm, melody, and improvisation, music therapy offers a scalable, engaging, and effective therapeutic approach. These findings support the integration of music-based interventions into early education and clinical settings, paving the way for more personalized and inclusive therapies for ASD.

## 1 Introduction

Autism spectrum disorder (ASD) is a complex neurodevelopmental condition associated with differences in social communication and interaction, along with restricted, repetitive patterns of behavior and sensory sensitivities (AlSalehi and Alhifthy, 2020; Faja and Dawson, 2017; Sharma et al., 2018). Globally, ASD affects approximately 1 in 44 children, with prevalence rates continuing to rise across socioeconomic and cultural contexts (Conner et al., 2024; Nedungadi et al., 2024). These characteristics may affect how individuals with ASD experience and navigate social relationships, emotional regulation, and new environments (Grossard et al., 2017; White et al., 2014). Such challenges not only affect the quality of life of individuals but also place a heavy emotional and economic burden on families and caregivers. In the United States alone, the lifetime cost for individuals with ASD is estimated to exceed $1.4 million, driven primarily by healthcare and educational needs (Buescher et al., 2014).

The increasing prevalence of ASD has spurred extensive research into effective intervention strategies. Early intervention has been identified as a critical factor for improving developmental trajectories in children with ASD (Darrou et al., 2010; Wetherby et al., 2018). This is because the early years represent a period of heightened neural plasticity, during which the brain is most receptive to environmental inputs (Kolb et al., 2017; Rogers S. J. et al., 2012). Numerous studies have shown that intensive early interventions can support the development of cognitive, language, and social capacities in autistic children, thereby empowering them to engage more confidently with their environments (Estes et al., 2015; Rogers P. P. et al., 2012). For example, early behavioral interventions have been linked to gains in IQ, adaptive functioning, and communication skills, demonstrating their potential to mitigate ASD-related challenges (Landa, 2018).

Among the most widely implemented early intervention strategies are Applied Behavior Analysis (ABA) and the Treatment and Education of Autistic and Related Communication Handicapped Children (TEACCH) program. ABA is based on the principles of operant conditioning and focuses on teaching specific behaviors through reinforcement and repetition (Lovaas, 1987; Malott and Kohler, 2021). Numerous meta-analyses have confirmed its effectiveness in improving language, social interaction, and adaptive behaviors in children with ASD (Makrygianni et al., 2018). For instance, ABA-based programs have been particularly successful in teaching children functional communication skills, such as requesting, labeling, and turn-taking (Schreibman et al., 2015).

Similarly, the TEACCH program emphasizes structured teaching and environmental adaptations to accommodate the unique cognitive and sensory profiles of individuals with ASD (Zeng et al., 2021). By creating highly organized and predictable environments, TEACCH aims to reduce sensory overload and anxiety, thereby promoting learning and independence. Research has shown that TEACCH can improve skills such as task completion, self-regulation, and social interaction, making it a valuable intervention for children with ASD (Odom et al., 2010).

Despite their documented benefits, these traditional interventions are not without limitations. ABA’s reliance on repetitive, task-based instruction can result in low engagement among children who lack intrinsic motivation or have sensory sensitivities (Keith, 2021; Lord et al., 2020). Furthermore, while TEACCH provides valuable structure, it may limit opportunities for spontaneous interaction and creativity, which are critical for fostering social and emotional growth (Lee et al., 2018; Odom et al., 2014; Thornhill-Miller et al., 2023). These limitations highlight the need for alternative or complementary approaches that prioritize engagement, flexibility, and holistic development.

Music therapy has emerged as a promising approach to supporting social engagement and emotional expression in autistic children (Marquez-Garcia et al., 2021). Unlike traditional behavioral interventions, music therapy leverages the universal appeal and multi-sensory nature of music to engage multiple brain systems simultaneously. Neuroimaging studies have shown that music activates the auditory cortex, motor planning areas, and limbic system, promoting neural integration and connectivity (Gordon et al., 2018; Pando-Naude et al., 2021). This multi-modal engagement creates a unique platform for fostering joint attention, improving emotional regulation, and enhancing social interaction, making music particularly well-suited for children with ASD (Lyu, 2024).

Empirical evidence supports the efficacy of music interventions in improving social communication, emotional responsiveness, and cooperative behaviors in children with ASD. Geretsegger et al. initially reported positive effects of music therapy on joint attention and social interaction in autistic individuals (Geretsegger et al., 2014). However, the updated Cochrane review rated the certainty of evidence for these outcomes as low to very low, indicating that further rigorous research is needed to confirm these effects (Geretsegger et al., 2022). Similarly, Sharda et al. reported that a 12-week structured music intervention improved verbal and social communication in autistic children, as measured by the Children’s Communication Checklist-2 (Sharda et al., 2018). However, the study did not include a follow-up assessment, so the durability of these effects over time remains unknown. Music’s inherent rhythm and melody provide a dual pathway for addressing both the cognitive and affective dimensions of social interaction. For example, rhythm-based activities, such as drumming and clapping, have been shown to enhance motor coordination and timing, which are critical for joint attention and cooperation (Woods, 2023). Meanwhile, melodic improvisation offers a non-verbal outlet for emotional expression, fostering deeper connections between children and their caregivers (Funahashi, 2022).

While existing studies underscore the general benefits of music therapy for ASD, several gaps remain. Most research has focused on the overall efficacy of music interventions without disentangling the specific contributions of distinct activities, such as rhythm training, interactive singing, or instrumental improvisation (Thaut and Hoemberg, 2014). This lack of specificity limits the ability of practitioners to tailor interventions to individual needs, potentially reducing their effectiveness. While Geretsegger et al. noted the lack of long-term evaluations, the updated Cochrane review includes more recent studies with extended intervention durations and follow-up assessments, thereby enhancing the applicability of findings (Geretsegger et al., 2014; Geretsegger et al., 2022).

Moreover, while neuroimaging studies have shed light on the mechanisms through which music influences brain connectivity, little is known about how individual differences—such as age, language ability, or sensory preferences—moderate the effects of music interventions (Zatorre and Salimpoor, 2013). Addressing these gaps is essential for advancing our understanding of music therapy and optimizing its implementation in clinical and educational settings.

The present study seeks to address these gaps by investigating the differential effects of distinct music activities on social skill development in preschool children with ASD. The intervention includes rhythm training, interactive singing, instrumental improvisation, and group games, each designed to target specific dimensions of social skills, such as communication, emotional regulation, and cooperation. By systematically comparing the effects of these activities, this research aims to identify which interventions yield the most significant improvements and explore the underlying mechanisms driving these effects.

In addition to its theoretical contributions, this study has practical implications for the design and implementation of music-based therapies. Understanding the specific mechanisms through which music activities improve social skills can help practitioners tailor interventions to the diverse needs of children with ASD, enhancing their efficacy and scalability. Furthermore, the findings may inform policy decisions, advocating for the integration of music therapy into early education and clinical frameworks.

Grounded in a neurodiversity-affirming framework, this study does not seek to “normalize” behavior but to expand opportunities for autistic children to express themselves, connect meaningfully with others, and participate in shared social experiences through music. This shift reflects a broader move in autism research from deficit-based models to relational, participatory approaches.

## 2 Methods

The intervention design and evaluation were grounded in a neurodiversity-informed framework, which recognizes autism as a variation in human neurocognitive functioning rather than a disorder to be corrected. All activities were designed to respect individual preferences and sensory profiles, aiming to enhance opportunities for mutual engagement, meaningful expression, and social connection.

### 2.1 Study design

This study adopts a randomized controlled trial (RCT) design to investigate the efficacy of therapeutic music intervention on the social skills development of preschool children diagnosed with autism spectrum disorder (ASD). The study aims to examine short-term and long-term outcomes of music-based activities and compare them with traditional behavioral interventions. The central concept, “Melodic Bridges,” emphasizes the role of music as a bridge to facilitate emotional, cognitive, and social connectivity, leveraging music’s unique properties to support social engagement and expression in young autistic children, within a neurodiversity-informed framework.

### 2.2 Participants

Participants in this study were preschool children aged 3 to 6 years who were diagnosed with ASD according to the Diagnostic and Statistical Manual of Mental Disorders, Fifth Edition (DSM-5). Recruitment was conducted through local preschools, specialized education centers, autism support organizations, and parent networks, ensuring a diverse sample that reflected real-world demographics. To enhance diversity, participants from different socioeconomic backgrounds were included. Parents provided official diagnostic documentation and behavior reports to confirm eligibility.

A total of 60 children were enrolled, who were randomly assigned to either the experimental group (*n* = 30) or the control group (*n* = 30) using stratified randomization. The sample size was determined based on *a priori* statistical power analysis for a two-sample *t*-test, assuming a significance level of 0.05, a power of 0.80, and an effect size (Cohen’s *d*) of 0.8 based on prior studies on similar interventions. This calculation indicated a minimum of 26 participants per group to detect a significant difference in social skill outcomes between groups. To account for an anticipated attrition rate of 10%, the initial sample size for each group was increased to 30 participants.

Stratification ensured balance between the two groups in terms of age, gender, and baseline social skills, measured using the Social Responsiveness Scale (SRS). The experimental group participated in therapeutic music intervention sessions, while the control group engaged in traditional behavioral interventions without music components.

Eligibility criteria for participants included: (1) being within the age range of 3 to 6 years; (2) meeting DSM-5 criteria for ASD, with observable differences in social interaction patterns; (3) possessing basic verbal communication skills (e.g., single words or short phrases); and (4) parental availability to support consistent attendance and provide feedback. Exclusion criteria were: (1) severe sensory impairments (e.g., vision or hearing loss) that could interfere with the intervention; (2) recent participation in similar music or behavioral interventions; (3) co-occurring conditions (e.g., epilepsy or severe anxiety); and (4) inability of the family to adhere to the intervention schedule or follow-up requirements.

Demographic information for all participants was collected prior to the intervention, including age, gender, socioeconomic background, and baseline social skills. The mean age of the sample was 4.5 years (SD = 1.1), and the gender distribution was balanced (50% male, 50% female). Baseline social skill scores on the SRS showed no significant differences between the experimental and control groups (*p* > 0.05), confirming initial equivalence.

To ensure ethical compliance, this study was reviewed and approved by the institutional ethics committee. Written informed consent was obtained from all parents or legal guardians after a thorough explanation of the study’s objectives, procedures, potential risks, and benefits. Participants’ privacy was safeguarded throughout the study, and all data were anonymized prior to analysis. Families were also informed of their right to withdraw from the study at any time without penalty.

No blinding was implemented in this study. Parents who completed the SRS and therapists who evaluated engagement were informed of group assignments. This limitation is acknowledged, and all raters were instructed to follow objective, behaviorally anchored criteria to reduce subjective interpretation.

### 2.3 Intervention procedure

Rather than aiming to normalize behavior or reduce autistic traits, the intervention was grounded in a participatory, neurodiversity-affirming approach that values each child’s unique communication style, sensory profile, and expressive potential. Music served as a platform for co-regulation and mutual engagement, allowing adults and peers to connect meaningfully with the child.

The intervention for this study consisted of a 12-week program, with sessions held three times per week, each lasting 60 min. The program was designed to leverage therapeutic music activities to improve specific social skills in preschool children with ASD. The intervention was divided into three key stages: warm-up activities, core activities, and a closing session, ensuring that the structure was suitable for maintaining the attention and engagement of young children.

The experimental group participated in music-based activities that emphasized interaction, emotional expression, and cooperation. Each session followed a standardized format:

#### 2.3.1 Warm-up activities (5 min)

The session began with greeting songs to establish a welcoming and predictable routine, followed by simple body movements to engage children, and prepare them for active participation.

#### 2.3.2 Core activities (45 min)

Singing: Interactive songs were chosen to encourage verbal participation and turn-taking. For example, call-and-response songs helped children practice social reciprocity.

Rhythm training: Simple percussion instruments, such as drums and tambourines, were used to promote joint attention and synchronization through group rhythm exercises.

Instrumental play: Children were encouraged to improvise using simple instruments (e.g., xylophones, maracas) to express emotions and foster creativity in a nonverbal social context.

Interactive games: Music-based group games, such as musical chairs or follow-the-leader, were included to build cooperation, shared activity participation, and peer interaction.

Closing activities (10 min): Each session concluded with reflective discussions and goodbye songs, reinforcing the day’s achievements and creating a sense of closure.

The control group participated in traditional behavioral interventions excluding musical elements, including structured teaching (TEACCH methods) and Applied Behavior Analysis (ABA). These interventions were implemented by licensed behavioral therapists who had received formal training in developmental behavioral protocols for children with ASD. Each therapist had a minimum of 2 years of field experience and was supervised by a senior behavioral interventionist to ensure treatment fidelity. Similar to the experimental group, two trained assistants supported each session by managing visual schedules and assisting with reinforcement systems.

All interventions were conducted in a child-friendly, acoustically optimized room equipped with age-appropriate instruments and materials. Sessions were facilitated by certified music therapists with at least 2 years of experience in ASD intervention. Therapists underwent specific training prior to the study to ensure consistency in implementing the intervention protocol. Additionally, two trained assistants supported each session by managing materials, observing children’s behaviors, and documenting session progress.

Individualized adjustments were made for children based on their baseline social skills, language abilities, and sensory preferences. For instance, quieter instruments were offered to children with auditory sensitivities, and additional verbal prompts were provided to those with limited language skills. These accommodations ensured that each child could fully engage in the activities without undue stress.

To monitor the fidelity of the intervention, a checklist was used during each session to document adherence to the standardized protocol. Therapists also held weekly debriefings to review challenges and adapt strategies as needed.

In this study, emotional regulation was operationally defined based on both behavioral observation and caregiver/teacher feedback. Observational indicators included not only the frequency of emotional expressions (e.g., frustration, withdrawal) but also the duration of recovery and the child’s ability to resume the task following emotional arousal. Each observed session was coded using a 5-point scale, where higher scores reflected shorter recovery time, increased use of self-soothing strategies (e.g., deep breathing, seeking support), and greater contextual appropriateness in emotional responses.

Meanwhile, qualitative feedback from caregivers and teachers was coded thematically, focusing on changes in the child’s ability to recognize, verbalize, and manage emotions over time. These qualitative insights were integrated with the quantitative observations to triangulate emotional regulation outcomes.

The intervention aimed to achieve three primary objectives: (1) support children’s ability to initiate and sustain reciprocal social interactions, (2) enhance cooperation in group settings, and (3) foster emotional expression and regulation through musical activities. By integrating sensory stimulation, emotional connection, and structured social learning, the music intervention was designed to serve as a bridge for developing essential social skills in children with ASD.

### 2.4 Outcome measures

To evaluate the effectiveness of the music intervention, a combination of standardized assessments, observational methods, and subjective feedback was employed. The outcome measures were designed to comprehensively capture changes in social skills and related domains in preschool children with ASD. Data were collected at four key time points: baseline (T0), mid-intervention (T1), post-intervention (T2), and follow-up (T3, 6 weeks after the intervention).

The primary outcome measure was the Social Responsiveness Scale (SRS), a widely validated parent-report questionnaire used to assess social communication profiles and interactional tendencies commonly associated with autism. The SRS includes subscales measuring social communication, social awareness, social motivation, and restricted interests, providing a comprehensive profile of social functioning. Scores were collected at all four time points to evaluate changes over time and differences between the experimental and control groups.

The secondary outcome measures included the following:

Naturalistic Observation: During sessions, trained observers documented specific social behaviors, such as initiating interactions, responding to peers, maintaining eye contact, and turn-taking. Observers used a predefined checklist to ensure consistency across sessions. Data were recorded during the core activities of intervention sessions at T1 and T2.

Emotion Regulation and Expression: Observers qualitatively evaluated children’s emotional responses during sessions, noting instances of frustration, excitement, or cooperation. This measure aimed to assess the indirect impact of the intervention on emotional regulation.

Parental and Teacher Feedback: Structured questionnaires were administered to parents and teachers at T2 and T3 to capture their perceptions of the children’s social skill development. Questions focused on changes in everyday interactions, such as playing with peers, responding to instructions, and participating in group activities.

Engagement Level: Therapists tracked each child’s level of engagement during sessions using a 5-point Likert scale, ranging from “not engaged” to “fully engaged.” This measure was collected at every session to monitor the children’s interest and involvement in the intervention.

To ensure the validity and reliability of the assessments, all observers and therapists underwent a one-week training program that included practice scoring using video examples and calibration exercises. Observers also conducted periodic inter-rater reliability checks throughout the study, with a target Cohen’s kappa value of 0.80 or higher to confirm scoring consistency.

Data quality control measures included double data entry to minimize errors and periodic reviews of observation logs. For subjective feedback, all questionnaires were anonymized and coded to reduce potential bias from parental expectations. The combination of standardized tools, direct observations, and subjective feedback ensured a robust evaluation of the intervention’s effects.

In alignment with a strengths-based and relational model of autism support, outcome measures were interpreted not as indicators of normalization, but as reflections of increased access to shared emotional and communicative experiences.

The study’s primary hypothesis was that the experimental group would demonstrate significant improvements in social skills, as reflected in SRS scores and naturalistic observation metrics, compared to the control group. Secondary analyses were conducted to explore whether specific music activities (e.g., rhythm training or singing) were associated with greater gains in certain aspects of social interaction, and whether these improvements persisted at follow-up (T3).

### 2.5 Data analysis

These analyses aimed to identify activity-specific patterns that facilitated social connection, rather than to evaluate the reduction of autistic traits.

All data were analyzed using SPSS version 26.0. The primary goal of the analysis was to evaluate the effectiveness of therapeutic music intervention on the social skills of preschool children with ASD compared to traditional behavioral interventions. Secondary analyses were conducted to explore the effects of specific music activities and the long-term sustainability of improvements. Statistical significance was set at *p* < 0.05 for all tests.

#### 2.5.1 Descriptive statistics

Descriptive statistics, including means, standard deviations, and frequencies, were calculated to summarize participants’ demographic characteristics and baseline social skills scores. Independent samples t-tests were used to ensure no significant differences existed between the experimental and control groups at baseline (T0) for key variables, such as Social Responsiveness Scale (SRS) scores.

#### 2.5.2 Primary analysis

To evaluate changes in SRS scores over time, a repeated measures ANOVA was conducted with time (T0, T1, T2, T3) as the within-subjects factor and group (experimental, control) as the between-subjects factor. The main effects of time and group, as well as the time × group interaction effect, were tested to determine whether the experimental group showed greater improvements over time compared to the control group. *Post-hoc* pairwise comparisons with Bonferroni correction were performed to identify specific time points with significant differences.

#### 2.5.3 Secondary analysis

For secondary outcome measures, the following analyses were conducted:

Naturalistic Observation Data: Independent samples t-tests compared the frequencies of observed behaviors between groups at T1 and T2. Repeated measures ANOVA was also used to examine changes within the experimental group over time.

Emotion Regulation: Changes in emotional regulation scores were analyzed using paired samples *t*-tests within each group and independent samples t-tests between groups.

Parental and Teacher Feedback: Data from feedback questionnaires were analyzed using Mann–Whitney *U* tests for non-normally distributed data or independent samples *t*-tests for normally distributed data.

Engagement Levels: Linear mixed-effects models were used to analyze session-wise engagement data across the 12-week intervention period, accounting for repeated measures and individual variability.

#### 2.5.4 Exploratory analysis

Exploratory analyses were conducted to examine the impact of specific music activities on SRS subscale scores. Pearson correlation coefficients were calculated to assess relationships between participation in specific activities and social skill improvements. A multiple linear regression model was used to explore predictors of long-term improvement at follow-up (T3), including baseline scores, engagement levels, and specific activity participation.

#### 2.5.5 Data quality control

All data were checked for normality using the Shapiro–Wilk test. For non-normally distributed data, appropriate non-parametric tests were employed. Missing data were handled using multiple imputation methods, ensuring that results remained robust. Inter-rater reliability for observational data was assessed using Cohen’s kappa, with values exceeding 0.80 indicating excellent agreement.

#### 2.5.6 Sensitivity analysis

To assess the robustness of the findings, sensitivity analyses were performed by excluding participants with incomplete data or those who withdrew from the study. Results were compared with the primary analysis to confirm consistency.

## 3 Results

### 3.1 Baseline characteristics

The baseline demographic and clinical characteristics of the experimental and control groups are presented in Table 1. The baseline demographic and clinical characteristics of the participants indicated no significant differences between the experimental and control groups. The mean age of children in the experimental group was 4.6 years (SD = 1.0), while the control group had a mean age of 4.4 years (SD = 1.2). Statistical analysis using an independent samples t-test confirmed no significant age difference between the groups (*p* = 0.56). Gender distribution was balanced across the two groups, with each group comprising 15 males and 15 females. A chi-square test for categorical variables showed no significant difference in gender proportions (*p* = 1.00). Baseline Social Responsiveness Scale (SRS) scores, which provide a profile of social communication and interaction tendencies associated with autism, were comparable between the groups. The experimental group had a mean SRS score of 78.2 (SD = 5.4), while the control group scored 77.6 (SD = 5.7). The t-test confirmed that there was no significant difference in SRS scores between the groups at baseline (*p* = 0.68). These results suggest that randomization successfully created equivalent groups at baseline, ensuring comparability and minimizing potential confounding effects in subsequent analyses.

**Table 1:** Baseline demographic and clinical characteristics of the experimental and control groups.

Variable	Experimental group (*n* = 30)	Control group (*n* = 30)	*p*-value
Age (years, mean ± SD)	4.6 ± 1.0	4.4 ± 1.2	0.56
Gender (Male/Female)	15/15	15/15	1.00
SRS score (mean ± SD)	78.2 ± 5.4	77.6 ± 5.7	0.68

*p*-values were calculated using independent samples *t*-test for continuous variables (age and SRS scores) and chi-square test for categorical variables (gender distribution).

### 3.2 Improvements in social skills: primary outcomes

The impact of the music intervention on the social engagement and communication profiles of preschool autistic children was assessed using the Social Responsiveness Scale across four time points: baseline (T0), mid-intervention (T1), post-intervention (T2), and follow-up (T3). The results, summarized in Table 2, demonstrate significant improvements in the experimental group compared to the control group in both total SRS scores and subscale dimensions.

**Table 2:** Improvements in SRS total and subscale scores across four time points.

Subscale	Time point	Experimental group	Control group	*p*-value	Effect size (d)
Social awareness	T0	15.2 ± 3.4	15.0 ± 3.5	0.85	–
	T1	12.5 ± 3.0	14.8 ± 3.3	0.02*	0.75
	T2	10.0 ± 2.5	14.5 ± 3.2	<0.001**	1.45
	T3	10.2 ± 2.7	14.6 ± 3.4	<0.001**	1.40
Social cognition	T0	18.1 ± 3.8	18.0 ± 3.9	0.92	–
	T1	15.5 ± 3.2	17.8 ± 3.7	0.03*	0.65
	T2	12.5 ± 2.9	17.2 ± 3.5	<0.001**	1.32
	T3	13.0 ± 3.1	17.5 ± 3.8	<0.001**	1.20
Social communication	T0	25.0 ± 4.5	25.2 ± 4.4	0.87	–
	T1	20.5 ± 4.0	24.8 ± 4.2	<0.01**	0.95
	T2	16.8 ± 3.8	24.0 ± 4.2	<0.001**	1.20
	T3	17.2 ± 4.0	23.5 ± 4.3	<0.001**	1.15
Social motivation	T0	20.3 ± 3.9	20.5 ± 4.0	0.78	–
	T1	16.0 ± 3.5	19.8 ± 3.8	<0.01**	0.85
	T2	12.1 ± 3.1	19.5 ± 3.8	<0.001**	1.34
	T3	12.5 ± 3.3	19.2 ± 3.9	<0.001**	1.30
Restricted interests and repetitive	T0	17.6 ± 4.0	17.4 ± 4.1	0.88	–
	T1	15.0 ± 3.5	16.8 ± 3.7	0.04*	0.60
	T2	12.0 ± 3.5	16.8 ± 3.7	<0.001**	1.10
	T3	12.3 ± 3.7	16.5 ± 3.8	<0.001**	1.05
Total SRS score	T0	78.2 ± 5.4	77.6 ± 5.7	0.68	–
	T1	66.5 ± 4.8	75.5 ± 5.6	<0.001**	1.55
	T2	62.0 ± 4.9	73.1 ± 5.5	<0.001**	1.65
	T3	63.0 ± 5.0	72.8 ± 5.7	<0.001**	1.60

*p*-values were calculated using repeated measures ANOVA with *post-hoc* Bonferroni correction. Effect sizes (Cohen’s d) were computed based on the mean difference between groups, divided by the pooled standard deviation. *p < 0.05; **p < 0.01.

The total SRS scores over four time points (T0, T1, T2, T3) highlight the potential of music intervention in enhancing social communication and interactional engagement among preschool children with ASD. At baseline (T0), there was no significant difference between the experimental and control groups (78.2 ± 5.4 vs. 77.6 ± 5.7, 𝑝 = 0.68), confirming the equivalence of groups established through randomization, as discussed in 3.1 Baseline Characteristics. By mid-intervention (T1), the experimental group showed a meaningful improvement in SRS scores, reflecting more adaptive social interaction profiles (66.5 ± 4.8), while the control group remained largely unchanged (75.5 ± 5.6, *p* < 0.001, 𝑑 = 1.55). At post-intervention (T2), the experimental group achieved a mean score of 62.0 ± 4.9, reflecting a 20% improvement from baseline. In contrast, the control group only showed a slight reduction to 73.1 ± 5.5 (*p* < 0.001, *d* = 1.65). These results suggest that the music intervention supported developmentally meaningful changes in social engagement over time. Notably, the experimental group maintained these gains at follow-up (T3), with a mean score of 63.0 ± 5.0 (*p* < 0.001), further underscoring the long-term impact of the music-based intervention.

A detailed analysis of the SRS subscales reveals that the intervention’s impact varied across different dimensions of social skills. The most significant improvements were observed in social motivation. Scores in the experimental group decreased from 20.3 ± 3.9 at T0 to 12.1 ± 3.1 at T2 (*d* = 1.34), representing a notable shift in motivational engagement with social partners. These improvements were sustained at T3, with scores of 12.5 ± 3.3. The control group exhibited minimal changes (*p* < 0.001). Scores improved significantly from 25.0 ± 4.5 at T0 to 16.8 ± 3.8 at T2 (*d* = 1.20). The follow-up scores (17.2 ± 4.0) remained significantly better than those in the control group (*p* < 0.001). Both subscales showed consistent improvement, with large effect sizes (*d* = 1.32 and *d* = 1.45, respectively). These improvements underscore the intervention’s capacity to support children’s responsiveness to social cues and foster joint attention. Although this subscale showed relatively smaller improvements, the experimental group achieved significant reductions at T2 (12.0 ± 3.5) and maintained similar scores at T3 (*d* = 1.10).

Mid-intervention results (T1) suggest that the effects of the music intervention began to emerge early, particularly in social motivation and communication. This early improvement may reflect the structured and interactive nature of the musical activities, which engaged key dimensions of interpersonal communication. While most gains peaked at post-intervention (T2), the experimental group successfully maintained these benefits at T3, highlighting the intervention’s potential for fostering long-term social skill development.

The large effect sizes (*d* > 1.0) across most subscales demonstrate not only statistical robustness but also developmental and relational relevance, particularly in domains associated with reciprocal interaction and social participation. The substantial improvements in social motivation and communication align with the intervention’s design objectives which emphasized active participation, turn-taking, and emotional expression through music.

### 3.3 Behavioral changes during intervention: secondary outcomes

The behavioral changes observed during the intervention underscore the multifaceted impact of the music-based program, as shown in Table 3. Positive shifts in engagement, emotional responsiveness, peer interaction, and collaborative group participation highlight the intervention’s capacity to support key aspects of social–emotional development in autistic preschoolers, respecting individual learning styles and communication preferences.

**Table 3:** Behavioral changes during intervention across four time points.

Behavioral indicator	Time point	Experimental group	Control group	*p*-value	Effect size (d)
Engagement level	T0	2.1 ± 0.8	2.2 ± 0.7	0.75	–
	T1	3.8 ± 0.6	2.5 ± 0.7	<0.001**	1.87
	T2	4.2 ± 0.5	2.7 ± 0.6	<0.001**	2.14
	T3	4.1 ± 0.5	2.6 ± 0.7	<0.001**	2.10
Emotional regulation	T0	2.5 ± 0.7	2.6 ± 0.8	0.82	–
	T1	3.5 ± 0.6	2.7 ± 0.7	<0.01**	1.33
	T2	4.0 ± 0.5	2.8 ± 0.6	<0.001**	1.89
	T3	3.9 ± 0.5	2.9 ± 0.7	<0.001**	1.75
Peer interaction frequency	T0	3.2 ± 1.0	3.1 ± 1.1	0.88	–
	T1	4.1 ± 0.9	3.2 ± 1.0	<0.01**	0.95
	T2	4.5 ± 0.7	3.3 ± 1.0	<0.001**	1.40
	T3	4.3 ± 0.8	3.2 ± 1.0	<0.001**	1.25
Compliance with rules	T0	3.0 ± 0.9	3.1 ± 0.8	0.76	–
	T1	4.0 ± 0.8	3.2 ± 0.9	<0.01**	1.08
	T2	4.5 ± 0.7	3.4 ± 0.8	<0.001**	1.58
	T3	4.4 ± 0.7	3.3 ± 0.9	<0.001**	1.50

*p*-values were calculated using repeated measures ANOVA with *post-hoc* Bonferroni correction. Effect sizes (Cohen’s *d*) were computed based on the mean difference between groups, divided by the pooled standard deviation. *p < 0.05; **p < 0.01.

Participant engagement levels significantly increased in the experimental group, rising from 2.1 ± 0.8 at T0 to 4.2 ± 0.5 at T2 (*p* < 0.001, *d* = 2.14). This growth was maintained at T3 (4.1 ± 0.5), reflecting the program’s capacity to cultivate sustained, intrinsically motivated participation in shared musical activities.

Emotional responsiveness and co-regulation improved significantly in the experimental group, particularly between T1 (3.5 ± 0.6) and T2 (4.0 ± 0.5). These gains were largely sustained at T3 (*p* < 0.001, *d* = 1.75), aligning with the intervention’s emphasis on emotional expression through interactive music-making.

The experimental group showed a marked increase in peer-oriented interactions, with scores rising from 3.2 ± 1.0 at T0 to 4.5 ± 0.7 at T2 (*p* < 0.001, *d* = 1.40). These gains were sustained at T3, suggesting enhanced reciprocal engagement and shared social attention among peers.

Participation in structured group routines improved steadily in the experimental group, peaking at T2 (4.5 ± 0.7, *p* < 0.001, *d* = 1.58) and remaining high at T3. These results suggest that music-based activities effectively supported cooperative turn-taking and mutual coordination in a group setting.

### 3.4 Emotional regulation and feedback from caregivers

These behavioral ratings were based not solely on the frequency of emotional expressions but incorporated observations of how children recovered from emotional episodes, such as their ability to self-soothe or re-engage with the task. For example, a child who initially reacted with frustration but quickly calmed down and resumed participation received a higher emotional regulation score than one who remained dysregulated.

The convergence of quantitative indicators and caregiver feedback highlights the intervention’s role in supporting emotional flexibility and co-regulatory behaviors, as shown in Table 4. These findings, consistent with improvements in primary and secondary outcomes, reinforce the intervention’s multifaceted benefits in enhancing social and emotional functioning among preschool children with ASD.

**Table 4:** Emotional regulation and feedback from caregivers.

Indicator	Time point	Experimental group	Control group	*p*-value	Effect size (d)	Key feedback themes
Emotional regulation	T0	2.5 ± 0.7	2.6 ± 0.8	0.82	–	Limited emotional expression and frequent frustration.
	T1	3.5 ± 0.6	2.7 ± 0.7	<0.01**	1.33	Increased ability to calm down during group activities.
	T2	4.0 ± 0.5	2.8 ± 0.6	<0.001**	1.89	Enhanced emotional stability and cooperative behavior.
	T3	3.9 ± 0.5	2.9 ± 0.7	<0.001**	1.75	Sustained emotional control and reduced frustration levels.
Caregiver feedback						
Cooperation in group tasks	T0	Limited	Limited	–	–	Significant improvement noted by teachers at T2.
	T3	Moderate	Limited	–	–	Cooperation improvements sustained per caregiver reports.
Peer Interaction	T0	Minimal	Minimal	–	–	Increased peer interaction observed at T2 and T3.
	T3	Moderate	Minimal	–	–	Improvements noted, particularly in shared play.

*p*-values were calculated using repeated measures ANOVA with *post-hoc* Bonferroni correction. Key feedback themes were derived from structured caregiver and teacher questionnaires. **p < 0.01.

Quantitative analysis of emotional regulation scores revealed significant improvements in the experimental group compared to the control group. At T0 (baseline), no significant difference was observed between the two groups (*p* = 0.82), but by T1 (mid-intervention), the experimental group showed notable improvements (3.5 ± 0.6 vs. 2.7 ± 0.7, *p* < 0.01, *d* = 1.33). These improvements continued to increase at T2 (post-intervention) with a mean score of 4.0 ± 0.5, suggesting greater emotional flexibility and increased capacity for co-regulation (*p* < 0.001, *d* = 1.89). At T3 (follow-up), the experimental group maintained these gains (3.9 ± 0.5), suggesting sustained benefits of the intervention.

Structured caregiver and teacher questionnaires revealed marked behavioral changes in emotional regulation among children in the experimental group. At baseline (T0), both caregivers and teachers reported heightened emotional expressions and emerging co-regulatory needs during transitions and structured activities. By T2, caregivers observed growing capacity in children to articulate and process emotions, particularly in recognizing and expressing frustration in socially meaningful ways. These developments were sustained at T3, with caregivers describing increased emotional awareness and more adaptive responses to challenging situations. Teachers also reported fewer disruptive incidents and improved emotional stability during group tasks.

In terms of social engagement, caregivers initially observed limited opportunities for reciprocal peer engagement. By T2, children began initiating and responding to social cues more frequently. Shared play, turn-taking, and cooperative activities became more common, especially in music-based group sessions. At T3, caregivers highlighted that these behaviors had extended to home environments, with children actively engaging with siblings and peers during unstructured play. Teachers corroborated these observations, emphasizing more active participation in shared activities and growing responsiveness to peer-initiated social cues. Large effect sizes (*d* > 1.0) across all time points underscore the intervention’s robust impact. Feedback from caregivers and teachers further validated these findings, providing a comprehensive view of the intervention’s efficacy.

Improvements in emotional regulation and related behaviors were already evident by T1, as both quantitative measures and qualitative reports began to indicate meaningful changes. By T2, these developments became more pronounced, with consistent alignment between observed behaviors and caregiver/teacher feedback. At T3, the persistence of these behavioral gains particularly in emotional attunement and collaborative participation—demonstrated the sustained effectiveness of the intervention.

### 3.5 Impact of specific music activities on social skills

Analysis of the distinct contributions of specific music activities revealed that each activity fostered engagement with different aspects of social interaction, communication, and emotional responsiveness. Table 5 illustrates the score improvements across SRS subscales for three activities: singing, rhythm training, and instrumental play.

**Table 5:** Correlations between music activities and SRS subscale improvements.

SRS subscale	Music activity	Correlation (r, *p*-value)
Social motivation	Rhythm training	(0.40, 0.03*)
	Instrumental improvisation	(0.40, 0.03*)
	Group games	(0.35, 0.05)
Social communication	Interactive singing	(0.52, <0.01**)
	Rhythm training	(0.30, 0.05)
	Group games	(0.38, 0.04*)
Emotional regulation	Instrumental improvisation	(0.45, 0.02*)
Social awareness	Rhythm training	(0.55, <0.01**)
	Interactive singing	(0.30, 0.05)
	Group games	(0.42, 0.03*)
Restricted interests and repetitive	Instrumental improvisation	(0.20, 0.10)

Correlation values (𝑟) represent the strength and direction of the relationship, with values closer to 1 indicating stronger positive relationships. Statistical significance is denoted by *p*, with *p* < 0.05 considered significant (*) and *p* < 0.01 highly significant (**). The “Restricted Interests and Repetitive Behavior” subscale showed no statistically significant correlations with the analyzed music activities.

Rhythm-based activities, such as drumming and clapping, were significantly associated with increased social orientation (Social Motivation: *r* = 0.40, *p* = 0.03∗) and awareness of others’ presence and actions (Social Awareness: *r* = 0.55, *p* < 0.01∗∗). These activities appear to support co-regulated attention and interactive rhythm synchronization, which may help build the foundations for mutual responsiveness. Structured rhythm-based activities, such as drumming and clapping, were particularly effective in fostering joint attention and turn-taking, aligning with the intervention’s design goals in 2. Methods. Additionally, a moderate correlation with Social Communication (*r* = 0.30, *p* = 0.05) indicates that rhythm training may also support basic verbal interaction.

Interactive singing showed the strongest correlation with social communication (*r* = 0.52, *p* < 0.01∗∗), particularly in supporting reciprocal vocal exchanges and shared musical dialogue. Moderate associations with Social Awareness (*r* = 0.30, *p* = 0.05) suggest that call-and-response formats may foster awareness of others’ emotional cues and timing in communication.

Instrumental improvisation was positively associated with emotional co-regulation (*r* = 0.45, *p* = 0.02∗), indicating that creative, nonverbal sound exploration can offer a safe platform for children to externalize and process affective states. A moderate correlation with Social Motivation (*r* = 0.40, *p* = 0.03∗) further suggests that improvisational play fosters intrinsic engagement and participatory expression.

Group games, such as “Follow the Leader” and “Musical Chairs,” showed moderate correlations with Social Motivation (*r* = 0.35, *p* = 0.05), Social Awareness (*r* = 0.42, *p* = 0.03∗), and Social Communication (*r* = 0.38, *p* = 0.04∗). These activities appeared to support coordination within shared routines, encourage turn-taking, and promote responsive peer engagement, particularly in structured musical contexts.

The SRS domain of restricted interests and repetitive behaviors showed no significant associations with any specific activity (e.g., *r* = 0.20 with improvisation, *p* = 0.10), suggesting that these patterns may not be readily modulated through the music-based strategies examined in this study. This highlights the importance of respecting individual sensory or behavioral preferences rather than targeting them for change.

## 4 Discussion

This study examined the effects of a 12-week participatory music intervention on the social and emotional engagement of preschool autistic children. Results demonstrated developmentally meaningful improvements in reciprocal communication, emotional responsiveness, and peer coordination. Rather than seeking to normalize behavior, this intervention leveraged music’s relational qualities to co-create environments in which autistic children could express, connect, and participate on their own terms.

Specific music activities contributed uniquely to different dimensions of social engagement. Interactive singing fostered reciprocal vocal exchanges and perspective-taking, aligning with neurocognitive research that links music to the activation of language and social cognition areas (Lemon-McMahon, 2019; Wang et al., 2024). Additionally, rhythm-based activities supported co-regulated attention and collaborative timing, echoing studies showing the link between rhythmic synchronization and joint social orientation (Keller et al., 2014). The improvement in emotional regulation, particularly through instrumental improvisation (*r* = 0.45, *p* = 0.02), is noteworthy. Instrumental improvisation provides a safe environment for emotional expression, allowing children to explore and regulate their emotions non-verbally. This finding supports the emotional resonance theory, which posits that music facilitates emotional attunement and self-regulation through shared auditory experiences (Molnar-Szakacs and Overy, 2006). Lastly, the moderate correlations observed for group games with social awareness (*r* = 0.42, *p* = 0.03) and cooperation reflect the importance of structured play in fostering peer interaction. These results align with developmental psychology theories that highlight the role of play in social learning (Cole and Scribner, 1978).

This work advances prior literature by mapping specific musical strategies onto unique patterns of social participation. While prior meta-analyses affirm the general utility of music therapy in autism (Ke et al., 2022), but few have dissected the relative impacts of rhythm, melody, and improvisation. Our results bridge this gap by showing that rhythm training excels in enhancing joint attention, while singing predominantly benefits verbal communication. The observed improvements also resonate with neurobiological studies. Music’s ability to engage both hemispheres of the brain may explain its multifaceted impact on social skills (Särkämö et al., 2016). For example, rhythm activates motor areas such as the basal ganglia, which are implicated in timing and coordination (Merchant et al., 2015), while melody engages the limbic system, enhancing emotional connectivity (Koelsch, 2014). In contrast to traditional behavioral therapies, which often rely on explicit instruction, music therapy leverages implicit learning through auditory and motor pathways. This may explain why children in the experimental group displayed sustained improvements at follow-up (T3), as music interventions capitalize on neuroplasticity, reinforcing social behaviors through repetitive, enjoyable activities (Thaut and Hoemberg, 2014).

The transformative effects of music can be attributed to its ability to engage multiple neural networks. Rhythm-based activities synchronize auditory and motor systems, promoting joint attention and coordination. Neuroimaging studies have shown that rhythmic synchronization strengthens connectivity between the auditory cortex and motor planning areas, such as the premotor cortex (Pranjić et al., 2024). This neural integration is critical for developing social behaviors, as it fosters turn-taking and mutual engagement. The emotional impact of music lies in its capacity to evoke and regulate emotions through limbic system activation. Instrumental improvisation engages the amygdala and anterior cingulate cortex, facilitating emotional expression and regulation (Ye, 2023). This aligns with the observed improvements in emotional regulation, suggesting that music provides a unique medium for children to process and manage their emotions in a safe, non-verbal context. Music also enhances social cognition by encouraging perspective-taking and empathy. Interactive singing, for instance, requires children to anticipate and respond to others’ vocal cues, activating regions involved in social cognition, such as the temporoparietal junction (Im et al., 2024; Ruiz Blais, 2023). This mechanism likely underpins the significant gains in social communication observed in this study.

Importantly, this intervention was grounded in a neurodiversity-affirming approach. We did not seek to eliminate autistic traits or enforce neurotypical norms. Instead, we supported alternative modes of connection, expression, and rhythm. The metaphor of “Melodic Bridges” symbolizes this shared space—where children, caregivers, and therapists meet in music, not to correct, but to attune. This resonates with recent calls to reframe autism research around respect, participation, and relational ethics (Bottema-Beutel et al., 2021; Lense and Camarata, 2020).

Practically, this suggests that music interventions can serve as adaptive, child-led complements to traditional behavioral programs. For children who struggle with structured verbal instruction, rhythm training may offer a more accessible entry point. For non-verbal children, improvisation may serve as a medium of affective expression. For children navigating peer engagement, singing-based exchanges can scaffold shared communication. Crucially, music’s intrinsic motivation, multi-sensory nature, and emotional resonance allow it to accommodate sensory and communicative diversity rather than override it.

This study also holds implications for educational policy and inclusive curriculum design. Music-based programs can be integrated into early education settings not as enrichment, but as core social–emotional learning tools. Because music does not depend on verbal language, it provides universal access to interaction, particularly for neurodivergent learners. Policymakers and educators should consider expanding access to certified music therapists and embedding participatory, non-directive music activities within classroom routines.

While this study provides compelling evidence for the efficacy of music interventions, several limitations warrant consideration. First, the relatively small sample size (*n* = 60) limits the generalizability of the findings. Second, the reliance on parent-reported measures, while practical, may introduce subjective bias. Future research should incorporate objective metrics, such as neuroimaging or physiological data, to validate the observed outcomes. Additionally, cultural, and individual differences in musical preferences were not explored, which may influence the intervention’s effectiveness. Expanding research to include diverse populations and exploring culturally adapted interventions will be crucial for broader applicability. Longitudinal studies are also needed to assess the durability of these effects and their impact on developmental trajectories. We acknowledge that the absence of blinding for both caregivers and therapists is a limitation of the study. This reflects a common challenge in applied interventions involving close adult-child interaction. Although standardized instruments were used, the possibility of expectancy effects influencing outcomes cannot be excluded. Future studies should address this by integrating third-party observers or double-coded behavioral recordings to validate findings. The integration of observational and caregiver-reported indicators of emotional regulation presented both strengths and limitations. While the multi-informant design offers a broader perspective, we acknowledge that the operational definition of emotional regulation in this study may not fully capture the construct’s complexity. Future research should incorporate more fine-grained coding systems (e.g., latency to recovery, quality of regulatory strategy) and use validated rating scales specific to emotion regulation to enhance construct validity.

In summary, this study highlights the capacity of music to act not as a corrective tool, but as a relational medium through which autistic children can express emotion, connect with others, and co-construct meaningful interaction. By embedding neurodiversity-informed values into the design and interpretation of intervention research, we move closer to building supports that respect and reflect the richness of autistic experience.

## Data Availability

The original contributions presented in the study are included in the article/supplementary material, further inquiries can be directed to the corresponding author.
